# Inflammatory Modulation Effect of Glycopeptide from *Ganoderma capense* (Lloyd) Teng

**DOI:** 10.1155/2014/691285

**Published:** 2014-05-22

**Authors:** Yan Zhou, Song Chen, Ran Ding, Wenbing Yao, Xiangdong Gao

**Affiliations:** State Key Laboratory of Natural Medicines, School of Life Science and Technology, China Pharmaceutical University, Nanjing, Jiangsu 210009, China

## Abstract

Glycopeptide from Ganoderma capense (Lloyd) Teng (GCGP) injection is widely used in kinds of immune disorders, but little is known about the molecular mechanisms of how GCGP could interfere with immune cell function. In the present study, we have found that GCGP had inflammatory modulation effects on macrophage cells to maintain NO production and iNOS expression at the normal level. Furthermore, western blot analysis showed that the underlying mechanism of immunomodulatory effect of GCGP involved NF-**κ**B p65 translation, I**κ**B phosphorylation, and degradation; NF-**κ**B inhibitor assays also confirmed the results. In addition, competition study showed that GCGP could inhibit LPS from binding to macrophage cells. Our data indicates that GCGP, which may share the same receptor(s) expressed by macrophage cells with LPS, exerted immunomodulatory effect in a NF-**κ**B-dependent signaling pathway in macrophages.

## 1. Introduction

*Ganoderma lucidum* (GL) is a traditional Chinese medicine known to contribute to various biological and medicinal functions including hypoglycemic, antitumour, carcinostatic, antimutagenic, anti-inflammatory, antiallergic, antiandrogenic, and antiviral properties [1–6]. Many investigators have shown interests in isolating and characterizing new functional compounds from* Ganoderma lucidum*, such as polysaccharides [7], terpenoids [8], polysaccharide-peptide complexes [9], and proteins [10]. Glycopeptides are constituted by an oligopeptide chain covalently attached to one or more carbohydrate moieties, and have a wide variety of biological functions. The functions may be dependent on the oligopeptide part, the carbohydrate part, or on both.

*Ganoderma capense *(Lloyd)Teng, called “Bozhi” in China, is the mycelium of Boshuzhi, a subgenus in* Ganoderma lucidum.* Bozhi glycopeptide injection is the sterile aqueous solution extract from dry mycelium powder of GCL, cultured by liquid fermentation method, containing 2.5 mg/mL polysaccharides and 0.5 mg/mL polypeptides. Bozhi glycopeptide injection has already been used clinically to treat progressive muscular dystrophy, myotonic dystrophy, dizziness, and vegetative nerve functional disturbance caused by vestibular dysfunction and high blood pressure and also aid in the treatment of cancer and hepatitis [11–13]. However, further studies are still needed to clarify the molecular structure and the molecular mechanism of Bozhi glycopeptide injection action. In our previous study, we have found that GCGP was composed of glucose with average molecule weight about 1 k, and the glycosidic linkage was (1 → 6) (1 → 2) linking ways. Also, the peptide chain of GCGP mainly contained glycine, alanine, valine, leucine, cysteine, aspartic acid, glutamic acid, serine, and threonine with O-glycopeptide linkage [14]. Several studies have reported that the immunomodulatory effect may play a key role in glycopeptides action [15, 16]. Macrophages play an important role in immune regulation, and we have also demonstrated that macrophages can mediate the effects of biologically active natural product isolated from fungi [17]. Previous studies have shown that Bozhi glycopeptide injection could activate mouse peritoneal macrophages [18]. In this study, we mainly focus on the inflammatory modulationeffects and mechanisms of the active compound glycopeptide GCGP on macrophage cells.

## 2. Materials and Methods

### 2.1. Reagents

Dulbecco's modified Eagle's medium was purchased from Gibco (Grand Island, NY, USA). Fetal bovine serum was from Sijiqing (Hangzhou, Zhejiang, China). Penicillin, streptomycin, and nuclear and cytoplasmic extraction kit were from Shenggong (Shanghai, China). Abs to phospho-I*κ*B-*α*, I*κ*B-*α*, NF-*κ*B-p65, *β*-actin, and JSH-23 were all from Santa Cruz (Santa Cruz, CA, USA). PDTC, MG-132, BAY 11-7082, BCA protein assay kit, nitric-oxide synthase assay kit, and abs to histone H3 were from Beyotime (Nantong, Jiangsu, China). Abs specific to mouse TLR4 was from eBioscience (San Diego, CA, USA). LPS, o-phenylenediamine, fluoresceinamine (FLA), and 1-cyano-4-dimethylaminopyridinium tetrafluoroborate (CDAP) were from Sigma-Aldrich (St. Louis, MO, USA). Enhanced chemiluminescence (ECL) substrate was from Thermo Scientific.

### 2.2. Cell Line and Cell Culture

RAW264.7, a mouse macrophage cell line, was obtained from the American Type Culture Collection (ATCC) and cultured in Dulbecco's modified Eagle's medium, containing 10% fetal bovine serum, 100 U/mL penicillin, and 100 *μ*g/mL streptomycin at 37°C in a 5% CO_2_ atmosphere.

### 2.3. NO Production Assay

Nitrite accumulation was analyzed by Griess reagent. Cells (1 × 10^5^ cells/mL) were dispensed into 96-well plates and stimulated with GCGP for 24 h. Supernatants were then collected and mixed with 0.5 vol Griess reagent (1% (w/v) sulfanilamide, 0.1% (w/v) N-(1-naphthyl)ethylenediamine dihydrochloride, and 2% (v/v) phosphoric acid) and incubated at room temperature for 10 min. Nitrite production was determined by comparing the absorbance at 540 nm.

### 2.4. iNOS Enzymatic Activity Detection

Cellular iNOS activity was measured by the conversion of L-arginine to NO by use of a nitric-oxide synthase assay kit (Beyotime Institute of Biotechnology).

### 2.5. Signaling Inhibition and Antibody Blocking Assay

Cells were pretreated with PDTC (50 *μ*M), MG-132 (25 *μ*M), BAY 11-7082 (50 *μ*M), and JSh-23 (40 *μ*M) at 37°C for 30 min prior to addition of GCGP. For antibody blocking, cells were pretreated with mouse anti-TLR4 (20 *μ*g/mL) or medium at 37°C for 2 h. NO production and iNOS enzymatic activity were detected 24 h later.

### 2.6. Western Blot Analysis

Cells were treated with medium or GCGP as described above. Nuclear and cytoplasmic extraction kit was used to collect cytoplasmic proteins after incubating cells for 30 min and nuclear proteins after incubating cells for 1 h. Equal amounts of proteins were resolved on 12% SDS-polyacrylamide gel, electrotransferred onto a PVDF membrane (Millipore, USA), and then incubated in TBST buffer (50 mM Tris, pH 7.6, 150 mM NaCl, and 0.05% Tween-20) containing 3% BSA at 37°C for 2 h. The membrane was subsequently incubated with mAbs against phosphor-I*κ*B-*α*, I*κ*B-*α*, NF-*κ*B-p65, *β*-actin, or histone H3 at 4°C overnight, followed by incubation with the corresponding secondary antibodies conjugated with horseradish peroxidase at 37°C for 1 h. The protein bands were finally visualized using an ECL system.

### 2.7. Fluoresceinamine Labeling of LPS

LPS was conjugated to FLA according to the CDAP-activation method as previously described with slight modifications [19]. In brief, 3 mg of CDAP was added to an aqueous solution containing 9 mg of LPS with gentle stirring and maintained at pH 9.0 for 2.5 min. The CDAP-activated LPS was then mixed with 0.6 mg of FLA (pH adjusted to 8.0) and incubated at room temperature overnight. Fluoresceinamine labeled LPS (fl-LPS) was separated from the excess free FLA with an Amicon Ultra-15 centrifugal filter unit (Millipore, Billerica, MA, USA). The FLA and LPS amounts in fl-LPS were, respectively, quantified by measuring absorbance at 490 nm and phenol-sulfuric acid assay.

### 2.8. Competitive Binding Assay

RAW264.7 cell suspensions at a density of 1 × 10^6^ cells/mL in PBS were incubated with a mixture of fl-LPS and GCGP (at serial concentrations) for 1 h on ice. After three washes, cells were examined on a FACSCalibur flow cytometer (BD Biosciences, San Jose, CA, USA) with a 488 nm laser excitation and a 530 nm emission filter. Data were acquired from a minimum of 10000 cells and analyzed using the FlowJo program (FreeStar, Ashland, OR, USA).

### 2.9. Statistical Analysis

For statistical analysis, GraphPad Prism software was employed. One-way ANOVA or *t*-test was used for determining the statistically significant differences between the values of various experimental groups. Data were expressed as means ± SD and a *P* value of 0.05 was considered statistically significant and of 0.01 was considered statistically very significant.

## 3. Results

### 3.1. Effect of GCGP on NO Production and iNOS Expression

**Figure 1:**
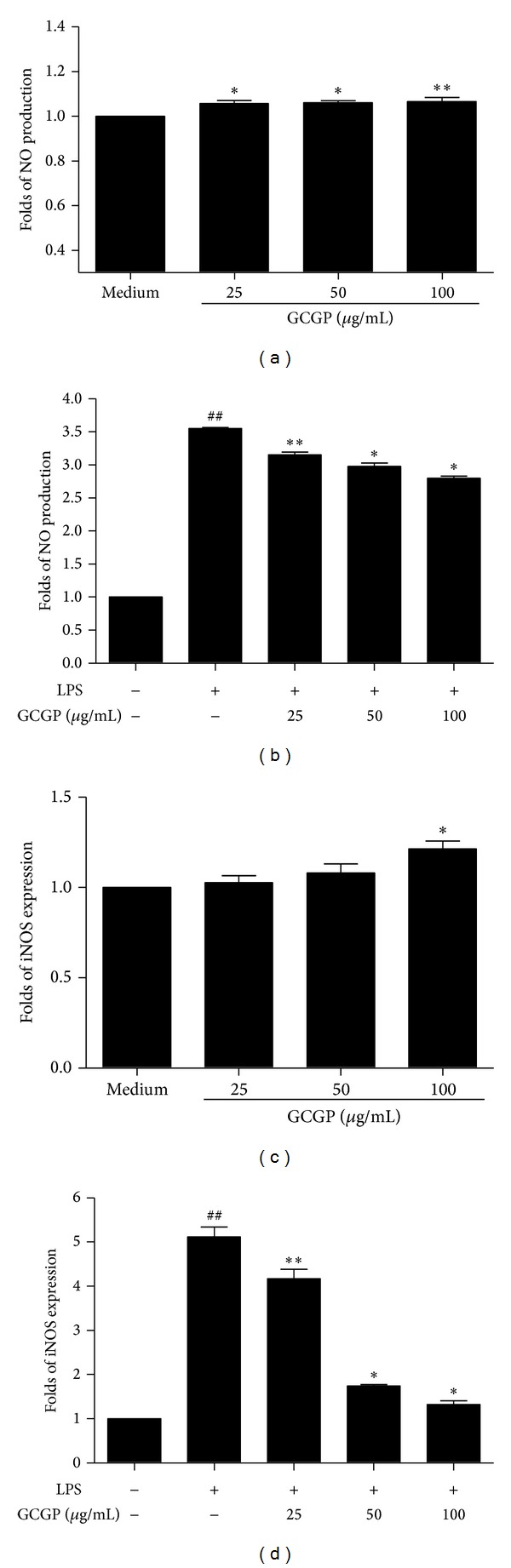
Effect of GCGP on NO production and iNOS expression in RAW264.7 cells ((a) and (c)). Cells were treated with the indicated concentrations of GCGP for 24 h ((b) and (d)). Cells were treated with the indicated concentrations of GCGP for 1 h prior to 5 *μ*g/mL LPS for 24 h. NO production and iNOS expression were analyzed as described in Materials and Methods. Results correspond to the mean ± SD of three independent experiments. ^##^ *P* < 0.01 versus control, *P < 0.05, and **P < 0.01 versus medium or LPS (ANOVA).

As shown in Figure 1, exposure of resting RAW264.7 cells to GCGP for 24 h resulted in dose-dependent increases in NO production (Figure 1(a)) and iNOS expression (Figure 1(c)) compared with untreated controls. LPS is one of the most powerful activators of macrophages. In this paper, activated RAW 264.7 cells were induced by LPS. RAW 264.7 cells were pretreated with different concentrations of GCGP for 1 h. It was found that LPS-induced NO production (Figure 1(b)) and iNOS expression (Figure 1(d)) were significantly decreased in a concentration-dependent manner.

### 3.2. Effects of GCGP on NF-*κ*B Activity

**Figure 2:**
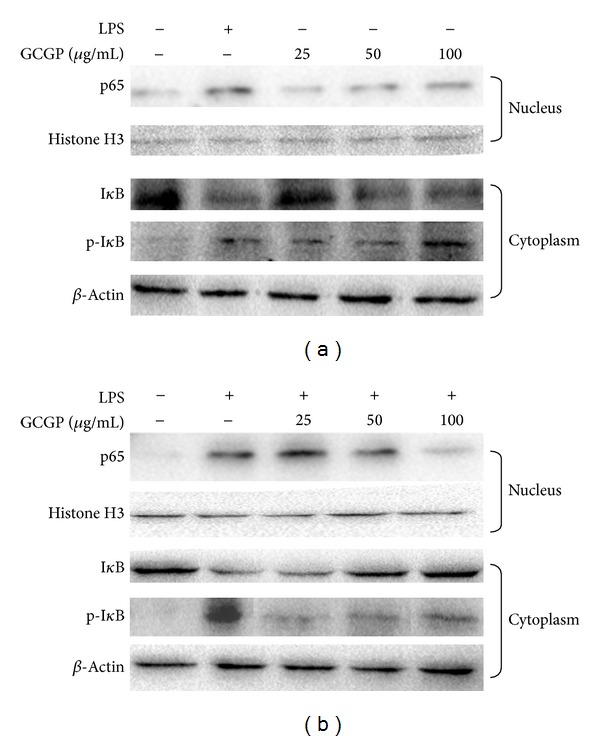
Effects of GCGP on NF-*κ*B activity in RAW264.7 cells. (a) GCGP activated NF-*κ*B signaling in normal RAW264.7 cells. (b) GCGP inhibited NF-*κ*B signaling in LPS-activated RAW264.7 cells.

As the activations of NO production and iNOS expression are critically required for the activations of NF-*κ*B, we determined the phosphorylation and degradation of cytoplasmic I*κ*B-*α* and translocation of NF-*κ*B subunits p65 from cytoplasm to nucleus. In our study, the I*κ*B-*α* protein underwent a significant phosphorylation and degradation at 30 min in the presence of GCGP, and NF-*κ*B-p65 subunit markedly translocated from the cytoplasm into the nucleus after stimulation by GCGP for 1 h (Figure 2(a)). Pretreatment with GCGP at different concentrations for 1 h, LPS-induced cytoplasmic I*κ*B-*α* phosphorylation, degradation, and NF-*κ*B-p65 translocation were reduced in a dose-dependent manner (Figure 2(b)).

### 3.3. Effects of NF-*κ*B Inhibitors on NO Production and iNOS Expression Induced by GCGP

**Figure 3:**
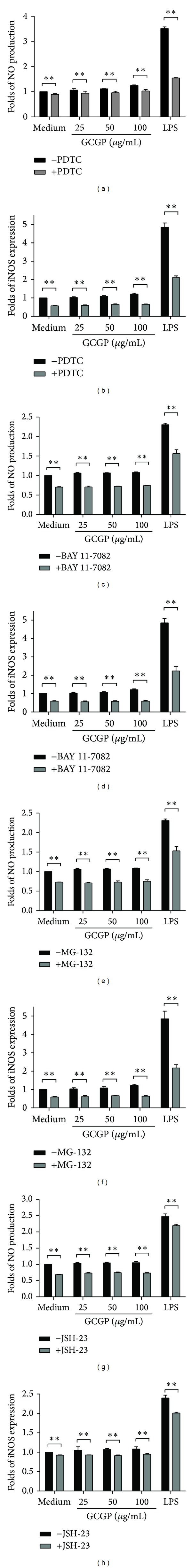
Effect of NF-*κ*B inhibitors on NO production and iNOS expression induced by GCGP. Cells were pretreated with 50 *μ*M PDTC ((a) and (b)), 50 *μ*M BAY 11-7082 ((c) and (d)), 25 *μ*M MG-132 ((e) and (f)), or 40 *μ*M JSH-23 ((g) and (h)) at 37°C for 30 min; then the indicated concentrations of GCGP were added for culturing 24 h. NO production and iNOS expression were analyzed as described in Materials and Methods. Results correspond to the mean ± SD of three independent experiments. **P < 0.01.

To further investigate the effect of GCGP on NF-*κ*B activation, NF-*κ*B inhibitors were involved. Pretreated cells with 50 *μ*M PDTC (an antioxidant), 50 *μ*M BAY 11-7082 (suppression phosphorylation of protein I*κ*B-*α*), 25 *μ*M MG-132 (a proteasome inhibitor, suppression degradation of protein I*κ*B), and 40 *μ*M JSH-23 (an inhibitor of NF-*κ*B transcriptional activity) for 30 min, NO production (Figures 3(a), 3(c), 3(e), and 3(g)), and iNOS expression (Figures 3(b), 3(d), 3(f), and 3(h)) activated by GCGP were obviously decreased.

### 3.4. Effects of GCGP Competitively Binding RAW264.7 Cells with FITC-LPS

**Figure 4:**
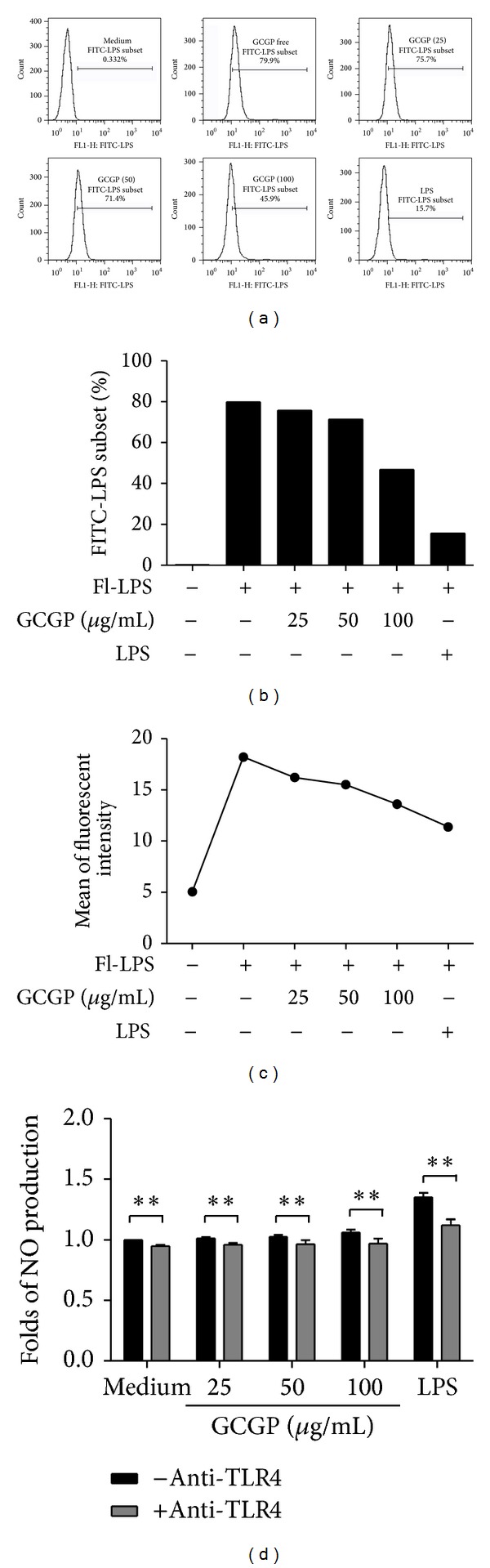
Effect of GCGP competitively binding RAW264.7 cells with FITC-LPS ((a) and (b)). Percent of positive FITC-LPS (FITC-LPS subset). (c) Mean of fluorescent intensity. Cells were incubated with 5 *μ*g/mL FITC-LPS and unlabeled GCGP (25, 50, and 100 *μ*g/mL) or unlabeled LPS (5 *μ*g/mL) together for 1 h at 4°C and then examined by flow cytometric analysis. (d) Anti-mouse TLR4 attenuated NO production induced by GCGP in RAW264.7 cells. Cells were pretreated with anti-TLR4 (20 *μ*g/mL) or medium for 2 h before the addition of GCGP. After 24 h incubation, NO production was analyzed as described in Materials and Methods. Results correspond to the mean ± SD of three independent experiments. **P < 0.01.

In competition assay, cells were incubated with FITC-LPS and unlabeled GCGP together for 1 h and then subjected to flow cytometric analysis. Unlabeled GCGP with different concentrations resulted in a dose-dependent decrease in the percent of positive FITC-LPS (FITC-LPS subset) (Figures 4(a) and 4(b)) and mean of fluorescent intensity (Figure 4(c)), suggesting that FITC-LPS binding to RAW264.7 cells can be competitively inhibited by GCGP. In addition, the requirement of TLR4 for GCGP function was investigated in RAW264.7 cells following receptor blocking with specific antibodies. Antibodies to TLR4 significantly reduced the NO production effect of GCCP, when compared with TLR4 antibody-free control (Figure 4(d)).

## 4. Discussion

The biologically active natural products, such as glycopeptides [14], proteoglycan [20], and polysaccharide [1], have attracted considerable attention due to their widely reported immune regulation and low toxicity. One of the approaches to evaluating immunomodulating activity of a natural substance is the assessment of its capacity of activating individual components of the immune system and promoting cytokine synthesis [15].

Macrophages are vital to the regulation of immune responses and the development of inflammation. As scavengers, they are highly specialized in removal of dying or dead cells and cellular debris. Along with dendritic cells, they play a crucial role in initiating immune response by presenting antigen. As secretory cells, they secrete a wide array of powerful cytokines, such as tumor necrosis factor (TNF) and interleukin-1 (IL-1). Meanwhile, they carry receptors for lymphokines that allow them to be “activated” into single-minded pursuit of microbes and tumour cells [21, 22]. Macrophages exposed to stimulating agents including lipopolysaccharides (LPS) can release several inflammatory cytokines and other substances. Nitric oxide (NO) is one of them, which can directly induce tumoricidal activity in macrophages. Lee et al. found that the Agrocybe chaxingu beta-glucan (polysaccharide) significantly inhibited LPS-induced NO levels in RAW264.7 cells, suggesting that this polysaccharide may be used for NO-related disorders such as inflammation [23]. Ji et al. found that the exposure of bone marrow macrophages to* Ganoderma lucidum* immunomodulating substance (GLIS) resulted in significant increases in NO production and then activated the immune system [24]. In our study, we have found that GCGP adjusted NO production in RAW264.7 cells, which is probably one of the mechanisms of its clinical effect.

NO has been proved to be a main effector molecule destructing tumor cells by activated macrophages [25]. It exerts multiple modulating effects on inflammation and is important in regulating immune responses. It virtually affects every step of the development of inflammation [26]. NO is a key vertebrate biological messenger, playing a key role in a variety of biological processes such as proliferation and development of Th1 cells [27]. Low level of NO promotes Th1 cells proliferation, and then Th1 cells activate macrophages by secreting IFN-*γ*. Interestingly, high concentration of NO indirectly limits the excessive activation of Thl cells by inhibiting the synthesis of IL-12. The dual role of NO in regulation of inflammation is also recognized. Different levels of NO production and the duration to NO exposure are detrimental factors in mediating the degree of inflammation and tissue injury [28]. Overproduction of NO is known to be associated with various diseases, such as cancer, rheumatoid arthritis, septic shock, autoimmune disease, and chronic inflammation [29]. The dual role of NO is important for maintaining immune homeostasis. In the present study, our results showed that GCGP can significantly induce NO production in RAW264.7 cells. Interestingly, as to high level of NO produced by LPS-activated RAW264.7 cells, GCGP could dose-dependently decrease the high NO production. NO is biosynthesized endogenously from L-arginine, oxygen, and NADPH by various nitric-oxide synthases (NOS), such as eNOS (endothelial NOS), nNOS (neuronal NOS), and iNOS (inducible NOS) among which eNOS and nNOS are constitutive (cNOS) and iNOS is inducible (iNOS) [30]. In macrophages, NO is synthesized mainly through iNOS [31]. In our study, we found that GCGP dose-dependently promoted iNOS expression in macrophage cells and inhibited iNOS expression in a dose-dependent manner in LPS-activated RAW264.7 cells. All these results suggested that GCGP has a dual role in maintaining immune homeostasis.

Nuclear factor kappa B (NF-*κ*B), a transcription factor, is known as an important regulator that controls the expression of various proinflammatory enzymes and cytokines and iNOS [32]. Normally, NF-*κ*B exists as an inactive form in the cytoplasm by binding to inhibitory kappa B (I*κ*B) protein [33]. Once I*κ*B protein is degraded, NF-*κ*B rapidly translocates into the nucleus, binds to the promoter elements of its targeted genes, and then regulates transcription of various genes including the downstream genes (such as iNOS, Cydin D1, and MMP9) [34]. As we had already found that GCGP can regulate NO production and iNOS expression in macrophage cells, consequently we considered whether GCGP exerted its action through a signaling pathway involving NF-*κ*B. Phosphorylation and degradation of I*κ*B-*α* and translocation of NF-*κ*B p65 subunit were measured by western blot analysis. The results showed that GCGP significantly elevated the level of I*κ*B-*α* phosphorylation, degradation, and p65 translocation compared with untreated control. Furthermore, we demonstrated that the specific NF-*κ*B inhibitor PDTC, BAY 11-7082, MG-132, and JSH-23 can almost abolish the effects of GCGP on the NO production and iNOS expression in macrophage cells. In summary, these findings led to the conclusion that GCGP is able to trigger NF-*κ*B signaling pathway in macrophage cells, which is essential for GCGP-mediated regulation of macrophage cell function. LPS induces NF-*κ*B signaling pathway activation after initiating signaling by directly binding to the membrane receptor, Toll-like receptor 4 (TLR4), and then regulates a group of gene expressions involved in innate immune and inflammatory responses [35]. And in our present study we have already demonstrated that GCGP can also activate NF-*κ*B signaling. These data prompted us to further study the mechanism involved and explore whether the membrane receptor of GCGP on macrophage cells shares some common molecule with LPS receptor. We conducted competition assay to analyze the effect of GCGP binding to RAW264.7 cells in membrane level. FITC-LPS binding to RAW264.7 cells can be competitively inhibited by GCGP. In addition, antibody blocking assay showed that TLR4 is functionally correlated with the stimulation of RAW264.7 cells by GCGP. The results suggested that TLR4 may also be the receptor of GCGP.

## 5. Conclusions

In conclusion, we have demonstrated the immunomodulating activity of GCGP in RAW264.7 macrophage cells. And GCGP, which shares some membrane receptor expressed by RAW264.7 cells with LPS, regulates NO production and iNOS expression through a signaling pathway involving NF-*κ*B in macrophage cells. Our findings supply the more clear mechanism of GCGP as an efficacious and safe biological response modifier and provide new evidence for therapeutic value of GCGP in the treatment of kinds of inflammatory disorders.
